# PLASMA BDNF/IRISIN RATIO ASSOCIATES WITH COGNITIVE FUNCTION IN OLDER ADULTS

**DOI:** 10.1093/geroni/igad104.2237

**Published:** 2023-12-21

## Abstract

**Objectives:**

To examine whether plasma biomarkers brain-derived neurotrophic factor (BDNF), irisin, clusterin and BDNF/irisin ratio (BIR) could differentiate people with mild cognitive impairment (MCI) from cognitively normal (CN) individuals, and to explore their relations with cognitive performance.

**Methods:**

We included 124 participants with MCI and 126 CN participants from a community-based aged and cognitive health cohort. Plasma BDNF, irisin and clusterin were measured, and BIR was calculated. Global cognition was evaluated with Montreal Cognitive Assessment. T-tests, logistic regressions, and linear regressions were used to explore the relations between plasma biomarkers and cognitive function.

**Results:**

The plasma levels of irisin, but not BDNF, was significantly different between MCI and CN groups. Higher irisin concentration was associated with increased probability for MCI (OR: 1.06, p = 0.004) after adjusting for covariates. By contrast, BDNF, but not irisin, was linearly correlated with cognitive performance (β = 0.14, p = 0.033). BIR values were positively correlated with cognitive performance (β= 0.14, p = 0.036), and significant differences on BIR values existed between MCI and CN groups. The MCI risk decreased by 53% (OR=0.47, p = 0.043) with each unit increase in BIR values after adjusting for covariates. Plasma BDNF and irisin concentrations increased with aging, whereas BIR values remained stable across the ages. No significant results of clusterin were observed in the above analyses.

**Conclusion:**

Plasma BIR is a potentially reliable indicator which not only reflects the odds of the presence of MCI but also directly associates with cognitive performance in the aged population.

